# Antigen-Specific CD4 T Cells Are Induced after Intravesical BCG-Instillation Therapy in Patients with Bladder Cancer and Show Similar Cytokine Profiles as in Active Tuberculosis

**DOI:** 10.1371/journal.pone.0069892

**Published:** 2013-09-11

**Authors:** Julia Elsäßer, Martin W. Janssen, Frank Becker, Henrik Suttmann, Kai Schmitt, Urban Sester, Michael Stöckle, Martina Sester

**Affiliations:** 1 Department of Urology, Saarland University, Homburg, Germany; 2 Department of Transplant and Infection Immunology, Institute of Virology, Saarland University, Homburg, Germany; 3 Boxberg Centre, Urological Group and Clinic Derout/Pönicke/Becker, Neunkirchen, Germany; 4 Urologicum Hamburg, Hamburg, Germany; 5 Department of Pathology, Saarland University, Homburg, Germany; 6 Department of Internal Medicine IV, Saarland University, Homburg, Germany; National Institute for Infectious Diseases (L. Spallanzani), Italy

## Abstract

Specific T cell immunity in patients with active tuberculosis is associated with a decrease in multifunctionality. However, it is unknown whether cytokine profiles differ in patients with primary infection and those with prior contact. We therefore used intravesical immunotherapy with attenuated live Bacille Calmette–Guérin (BCG) in patients with urothelial carcinoma as a model to characterise the induction of systemic immunity towards purified protein derivate (PPD) and to study whether cytokine profiles differ depending on pre-existing immunity. Eighteen patients with non-muscle invasive bladder cancer were recruited during the BCG-induction course. Fifty-four healthy individuals served as controls. Interferon (IFN)-γ and interleukin (IL)-2 producing PPD-specific CD4 T cells were analysed longitudinally before each instillation using a rapid flow-cytometric whole blood immunoassay. Baseline levels of IFN-γ producing PPD-specific T cells were comparable to controls. T cells showed a 5-fold increase to 0.23% by week 2/3, and further increased 8-fold by week 4/5 (to 0.42%, p=0.0007). Systemic immunity was induced in all patients, although the increase was less pronounced in patients with pre-existing immunity. As in active TB, cytokine profiling during therapy revealed a lower percentage of multifunctional IFN-γ/IL-2 double-positive T cells compared to controls (60.2% vs. 71.9%, p=0.0003). Of note, when comparing patients with and without pre-existing immunity, cytokine profiles in patients with primary immunity were shifted towards IL-2 single producing T cells (p=0.02), whereas those in patients with pre-existing immunity were shifted towards IFN-γ single-positivity (p=0.01). In conclusion, systemic T cell responses were induced after BCG-therapy, and their kinetics and cytokine profile depended on pre-existing immunity. Decreased functionality is a typical feature of specific immunity in both patients with active tuberculosis and BCG-therapy. Among patients with active infection, a shift towards IL-2 or IFN-γ single-positive cells may allow distinction between patients with primary infection and cases with boosted immunity after prior contact, respectively.

## Introduction

Since 1976, immunotherapy with live bacille Calmette Guérin (BCG) has proven to be an effective adjuvant intravesical treatment to prevent progress and relapse after transurethral resection of nonmuscle invasive bladder cancer [1,2]. The antitumor effect of BCG-therapy is largely attributed to the induction of a strong innate immune response [3] followed by infiltration of T cells into the bladder [4,5]. This is predominantly characterized by a T-helper type response [6,7], and its extent was shown to correlate with clinical response [8]. Although the therapeutic activity is restricted to the bladder, mouse models indicate that live bacilli enter bladder-draining lymph nodes where T cell priming is initiated [9]. This suggests that local BCG-instillation may be associated with a systemic induction of specific T cells, although evidence on their induction kinetics and functional properties in humans is limited [10]. Interestingly, studies in the mouse revealed that T cell infiltration after BCG-instillation is more rapidly observed if animals were pre-immunised with BCG-vaccination, which suggests that a pre-existing immunity may be beneficial in accelerating T cell induction and hence therapeutic effect. This was supported by the observations that pre-existing immunity was associated with improved anti-tumor response after BCG-instillation in both mice and patients [9]. Thus, subcutaneous immunisation before instillation may represent a new therapeutic strategy to improve treatment outcome. In this context, the availability of rapid assays to assess the quantity and functionality of specific immunity on an individual basis is an essential prerequisite to monitor such strategies [11].

Estimations on the presence of systemic immunity towards BCG may be obtained using the tuberculin skin-test that detects primed T cells towards mycobacterial antigens as a delayed type hypersensitivity reaction [12]. Purified protein-derivate (PPD), the antigen used in skin-testing, is an extract of various mycobacterial proteins present in different mycobacterial species, including BCG. Skin-testing has some limitations as a monitoring tool as it often yields falsely negative results [13], and may cause boosting reactions that are not distinguishable from the dynamic changes induced by the instillation [14]. In recent years, *in vitro*-assays have been developed that allow a more detailed quantitation of *M. tuberculosis*-specific T cells. Most *in vitro*-assays may be performed from small volumes of whole blood within one day, are logistically less demanding, and allow serial testing and functional characterisation of antigen-specific T cells [15]. In the setting of infection with *M. tuberculosis*, assessment of functionality by cytokine profiling has already been used to distinguish patients with and without active disease [16–21]. Interestingly, a decrease in multifunctionality and a shift towards cytokine single-producing cells was recently shown to be a typical feature of antigen-specific CD4 T cells in patients with active tuberculosis [16–18]. Interestingly, among patients with a decreased percentage of IFN-γ/IL-2 double-positive cells, some showed a shift towards cells expressing IL-2 whereas others exhibited an IFN-γ single-positive status [16]. The reason for this dichotomy is currently unclear but may be related to the presence or absence of pre-existing immunity prior to the development of active tuberculosis.

Intravesical BCG instillation therapy may be considered as a model to study the induction of mycobacterial immunity after defined antigen contact in humans. We therefore have used a flow-cytometric whole blood assay to longitudinally characterise the induction kinetics and functionality of systemic PPD-specific T cells in patients before and after BCG-instillation. As pre-existing immunity towards PPD may impact the kinetics and functional profile of specific T cells after challenge, a particular emphasis was placed on the characterisation of the induction of specific immunity in patients with and without evidence for prior sensitisation with mycobacterial antigens.

## Materials and Methods

### Ethics statement

The study was approved by the local ethics committee (Ärztekammer des Saarlandes) and all patients gave written informed consent.

### Patients

The study was conducted among 18 bladder cancer patients who received BCG-instillation therapy for the first time (mean age 66.7±10.9 years). All individuals underwent transurethral resection of all visible tumors. Patient and tumor characteristics are shown in table 1. All patients were analysed during a standard induction course consisting of six weekly intravesical BCG-instillations (BCG-medac, RIVM strain derived from strain 1173-P2), and followed clinically according to current guidelines [22]. In two patients, the third and fifth instillation was delayed for 2 and 1 week due to nitrite positive urinary tract infection and logistical reasons, respectively. Blood was drawn before BCG-therapy as well as 1, 2 or 3 and 4 or 5 weeks thereafter, in each case before instillation. Maintenance therapy was given in 7 individuals according to center practice. Tumor recurrence or progression was defined on the basis of biopsy and urine cytology. Fifty-four age-matched healthy individuals (mean age 65.3±11.0 years, 45 males, 9 females) who underwent analyses for latent infection with *M. tuberculosis* without clinical suspicion of active disease were recruited as controls.

**Table 1:** Demographic and clinical characteristics of patients treated with Bacille Calmette–Guérin (BCG).

Demographic characteristics	Patients (n=18)
Years of age, mean±SD	66.7±10.9
Sex, male/female	15/3
Tumor characteristics*	
Single tumor	9/18 (50.0%)
Multiple tumor	9/18 (50.0%)
pTa	9/18 (50.0%)
pT1	6/18 (33.3%)
CIS	3/18 (16.6%)
G2/G3-grade tumors	15/18 (83.3%)
G2	11/15 (73.3%)
G3	4/15 (26.6%)
Low/high grade tumors	14/18 (77.8%)
Low grade	9/14 (64.3%)
High grade	5/14 (35.7%)
Associated CIS	0/18 (0%)
Recurrent tumor	9/18 (50.0%)
Perioperative mitomycin treatment	5/18 (27.7%)
Days to therapy, mean±SD	34±25
Non responders	8/18 (44.4%)
Months to relapse, mean±SD	7.5±5.9

* Ta/T1 - papillary tumors; CIS - carcinoma in situ; SD – standard deviation.

### Quantitation of PPD-specific CD4 T cells from whole blood

Characterisation of PPD-specific CD4 T cells was performed directly from heparinized whole blood for a total of 6h according to an established standard operating procedure as previously described [23]. Cells were stimulated with PPD (7.32 µg/ml, Tuberkulin for *in vitro* use (RT-50); Statens Serum Institute, Copenhagen, Denmark). Treatment with diluent (PBS) and with 2.5µg/ml *Staphylococcus aureus* Enterotoxin B (SEB, Sigma, Deisenhofen, Germany) served as negative and positive controls, respectively. Each stimulatory reaction was performed from 300µl blood in the presence of 1µg/ml anti-CD28 (clone L293) and 1µg/ml anti-CD49d (clone 9F10; BD, Heidelberg, Germany). For the last 4h incubation time (37°C, 5% CO_2_) 10 µg/ml Brefeldin A (Sigma, Deisenhofen, Germany) was added to accumulate cytokines intracellularly. Subsequently, erythrocytes were lysed and leucocytes were fixed using 10ml BD lysing solution/ml whole blood. Staining was performed using anti-CD4 (clone SK3), anti-IFN-γ (clone 4S.B3), anti-IL-2 (clone MQ1-17H12), and anti-CD69 (clone L78, all from BD). At least 15.000 CD4 T cells were analyzed on a FACS Canto II (BD) using Diva Software. The percentage of specific T cells was calculated by subtracting the frequency obtained by the respective control stimulation. The lower limit of detection was 0.05% CD4 T cells as previously established and applied in other studies of mycobacterial T cell immunity [16,23–25].

### Statistical analysis

Statistical analysis was carried out using GraphPad Prism-5.03 (La Jolla, Ca.). Differences between two groups were determined by Mann-Whitney-test. The Friedman-test was applied for longitudinal analysis. To compare PPD-status between two or more groups the fisher’s or chi-square test was applied.

## Results

### PPD-specific CD4 T cell immunity prior to therapy does not differ between patients and healthy individuals

**Figure 1:**
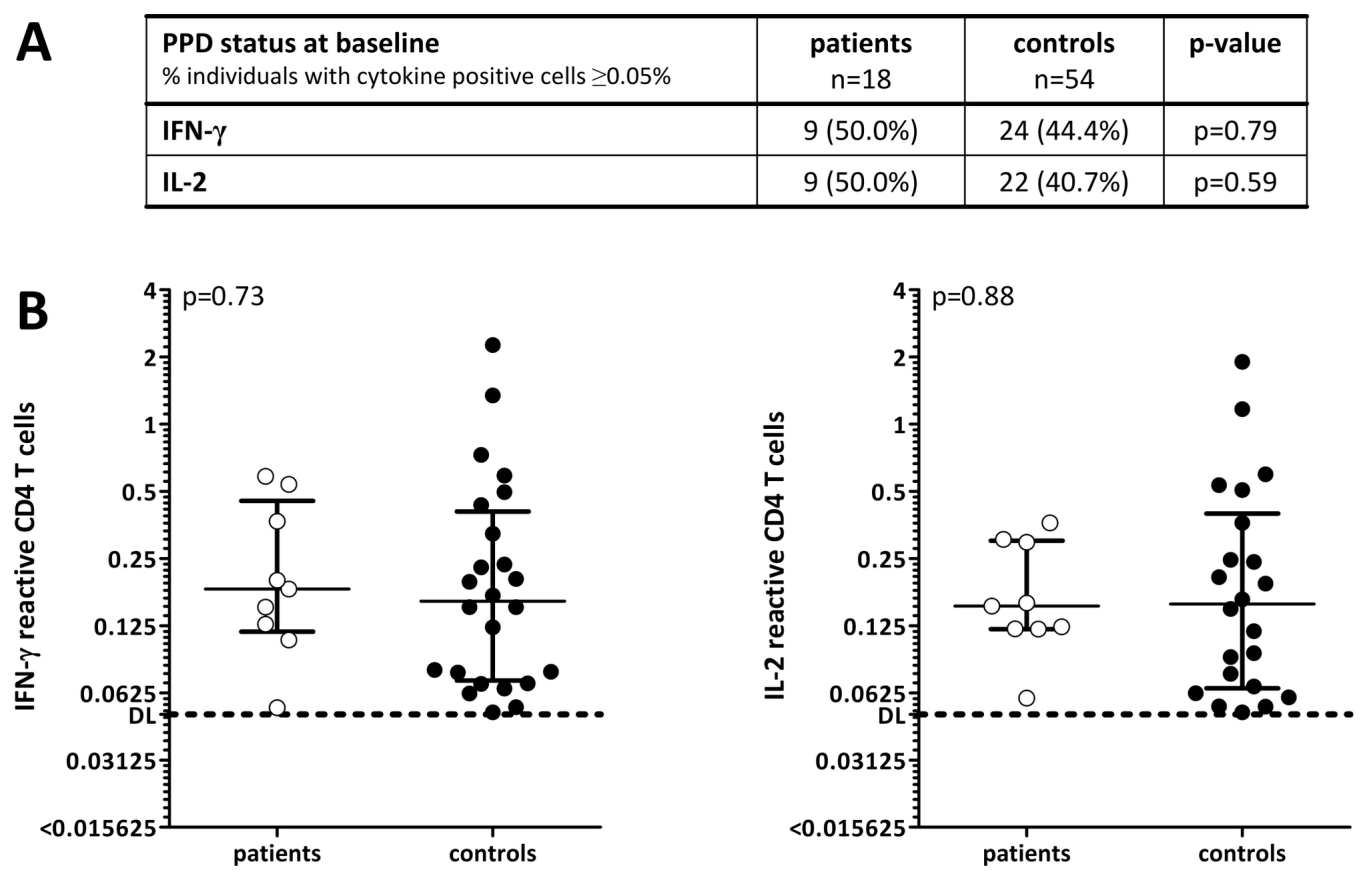
Bladder cancer patients before BCG-therapy and healthy controls do not differ in their baseline PPD-reactivity. (**A**) The percentage of individuals with pre-existing immunity towards PPD (≥0.05% CD69 positive interferon-γ (IFN-γ) or interleukin 2 (IL-2) producing CD4 T cells) and (**B**) PPD-reactive T cell frequencies of IFN-γ or IL-2 producing CD4 T cells were quantified in bladder cancer patients before BCG-therapy (n=18) and in age-matched healthy controls (n=54). In one patient, the baseline value was not available. However, as this patient did not have any specific immunity one week after start of therapy, baseline immunity was considered below detection limit. T cell frequencies of each individual (circles) as well as the median value and interquartile range (IQR) are shown, and the detection limit (0.05% reactive CD4 T cells) is represented by a dotted line.

To characterise the extent of pre-existing cellular immunity towards PPD, baseline frequencies of PPD-reactive CD4 T cells from 18 patients were compared to those of 54 age-matched controls. Antigen-specific CD4 T cells were identified based on the induction of the activation marker CD69 and the cytokines IFN-γ and IL-2 after PPD-stimulation. Although CD4 T cell frequencies elicited with the negative control stimulus were generally very low (median 0.008%, IQR 0.016%), the individual percentage of PPD-specific CD4 T cells was calculated by subtracting the frequency obtained by the respective control stimulation. In one patient, the baseline value was not available. However, as this patient did not have any specific immunity one week after start of therapy, baseline immunity was considered below detection limit. As exemplified for cells producing IFN-γ, 9/18 patients (50.0%) had PPD-specific T cells above detection limit. This was comparable to controls, where PPD-specific T cells were detectable in 24/54 individuals (44.4%, p=0.79, Figure 1A). Similar results were found when patients were stratified with respect to IL-2 producing PPD-specific T cells (Figure 1A), which were 88.9% concordant with IFN-γ positivity. When quantitative analyses were performed in individuals with PPD-reactive CD4 T cell frequencies above detection limit, there was no difference in median frequencies of IFN-γ or IL-2 producing PPD-reactive T cells in both groups (IFN-γ: patients 0.183%, IQR 0.399% versus controls 0.161%, IQR 0.355, p=0.73,; IL-2: patients 0.153%, IQR 0.241% versus controls 0.156%, IQR 0.347, p=0.88, Figure 1B). Thus, pre-treatment immunity towards PPD in patients shows a distribution that is comparable to healthy controls.

### Intravesical BCG-application induces a systemic T cell response towards PPD

**Figure 2:**
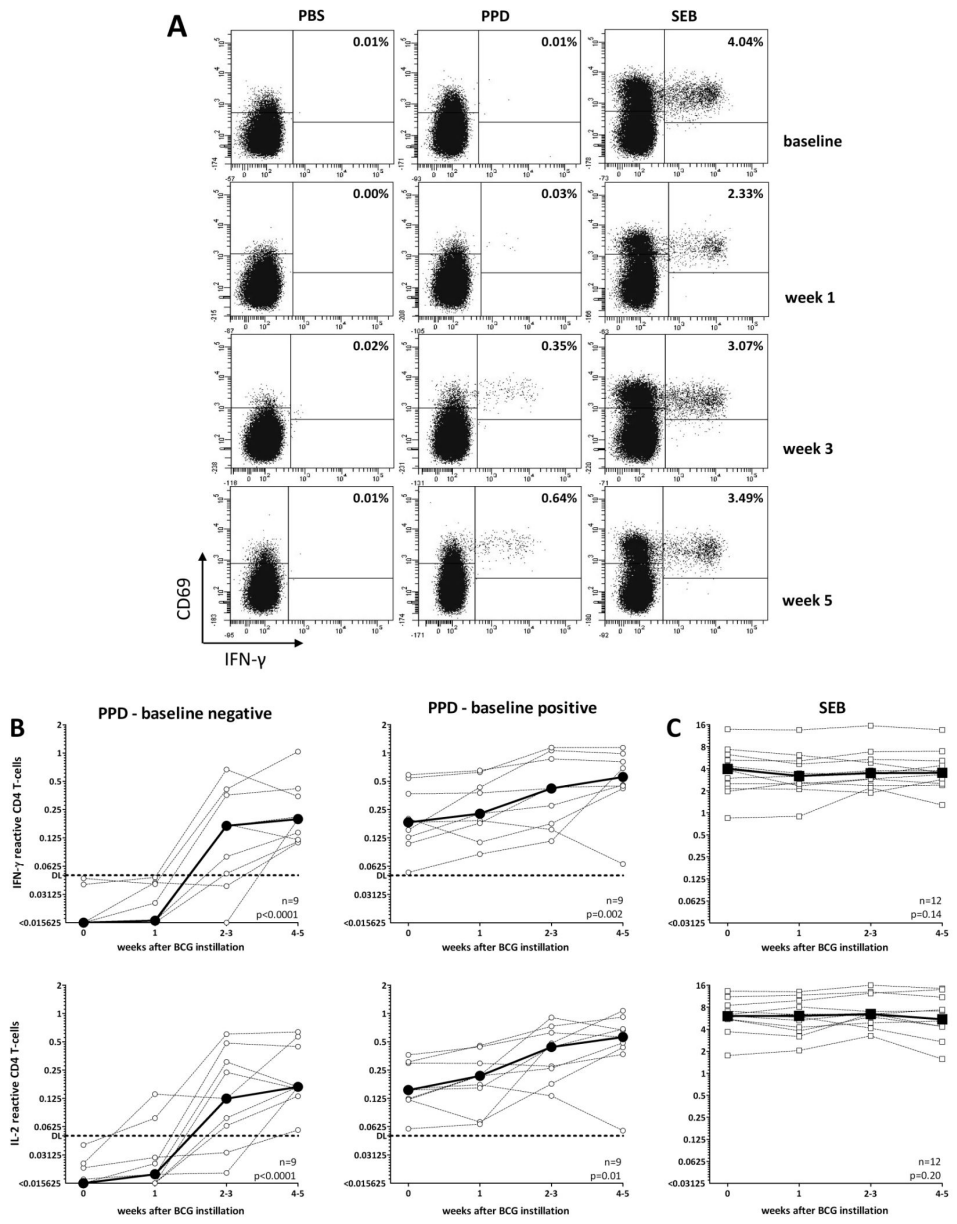
BCG-therapy induces a significant increase in systemic PPD-specific CD4 T cell immunity after the 2^nd^ instillation. (**A**) Representative examples of the kinetics of IFN-γ positive CD69/CD4 T cells before as well as 1, 3 and 5 weeks after the start of BCG-therapy. In this patient, the third instillation was delayed by 2 weeks due to urinary tract infection without fever. Numbers in the upper right quadrant refer to the percentage of CD69/IFN-γ positive cells among all CD4 T cells after stimulation with PPD, PBS (negative control), or *Staphylococcus aureus* Enterotoxin B (SEB, positive control). Results on IL-2 producing cells in this patient from the same stimulatory reaction are included in Figure 4A. Kinetics in the percentage of (**B**) PPD-specific or (**C**) SEB-reactive CD4 T cells producing IFN-γ or interleukin-2 (IL-2) of all patients during the induction course. Induction of PPD-reactive T cells is separately displayed for patients with and without detectable immunity at baseline. All patients were studied for PPD-specific T cells, and 12 patients were longitudinally analysed for SEB-reactive CD4 T cells (6/18 patients had a missing baseline value). Dotted lines represent kinetics of each patient, and repeated measurement from one patient after 2 or 3 and 4 or 5 weeks has been grouped as mean values. The bold line indicates the median percentage of reactive CD4 T cells.

**Figure 3:**
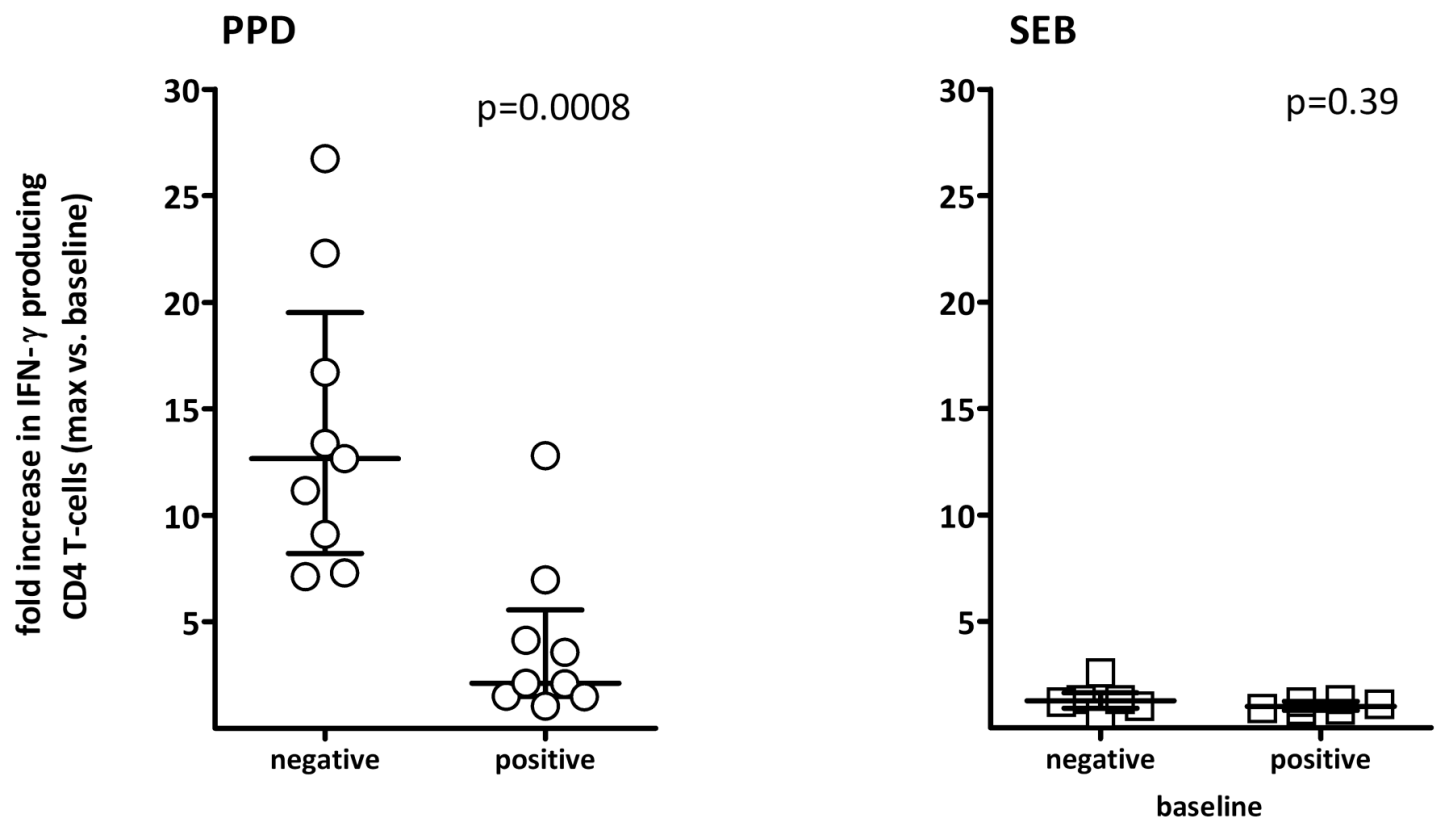
Higher increase in PPD-reactive T cell frequencies in patients without pre-existing immunity before therapy. The increase of PPD and SEB-reactive CD69 positive IFN-γ producing T cells between baseline and the maximum value during BCG-instillations was quantified for all patients and results were stratified for patients with negative and positive PPD-status before therapy. Medians and interquartile ranges are indicated.

The induction of PPD-specific immunity was then assessed before as well as 1, 2 or 3 and 4 or 5 weeks after the first instillation. Dotplots of PPD-specific T cells producing IFN-γ derived from a typical patient are shown in Figure 2A. While this patient did not show any specific immunity at baseline and one week after instillation, a strong PPD-specific T cell response was detectable one week after the 2^nd^ instillation (week 3) and T cell levels further increased in week 5. In contrast, SEB-reactive T cells were readily detectable and frequencies were largely stable over time. These findings were confirmed when longitudinally analysing PPD-specific T cells from all 18 patients. PPD-reactive T cell frequencies did not change within the first week. However, median frequencies of IFN-γ producing PPD-specific T cells showed an approximately 5-fold increase from 0.05% at baseline to 0.23% at week 2/3, and further increased 8-fold after week 4/5 (median 0.42%, p=0.0007, data not shown). Interestingly, when stratifying patients with and without pre-existing immunity, the increase in PPD-reactive T cells after BCG-instillation was significantly stronger in individuals where baseline levels were below detection limit, which held true for both IFN-γ and IL-2 producing T cells (Figures 2B and 3). However, maximum frequencies were significantly higher in patients with pre-existing immunity (p=0.02, data not shown). Of note, SEB-reactive T cells were detectable throughout the observation period, and their frequencies did not change over time (Figure 2C). When quantifying the percentage of individuals who acquired PPD-specific immunity over time, IFN-γ producing T cells after stimulation with PPD were detectable in 16/18 patients (88.9%) by week 2/3, and in all patients by week 4/5 (p=0.0004, table 2). This also held true when IL-2 producing T cells were quantified (p=0.0013, table 2). Taken together, a systemic induction of cellular immunity towards PPD may be quantified directly from whole blood after BCG-instillation, and PPD-specific immunity was induced in all patients.

**Table 2:** Percentage of individuals with a positive immune response above the detection limit of 0.05% towards PPD before and after BCG-instillation.

**week of therapy**	**baseline**	**1**	**2-3**	**4-5**	
Total number of patients	18	18	18	18	
IFN-γ positive (%)	9 (50.0%)	9 (50.0%)	16 (88.9%)	18 (100.0%)	p=0.0004
IL-2 positive (%)	9 (50.0%)	11 (61.1%)	16 (88.9%)	18 (100.0%)	p=0.0013

### Cytokine profiles of PPD-specific T cells after BCG-instillation differs from that observed in healthy controls

**Figure 4:**
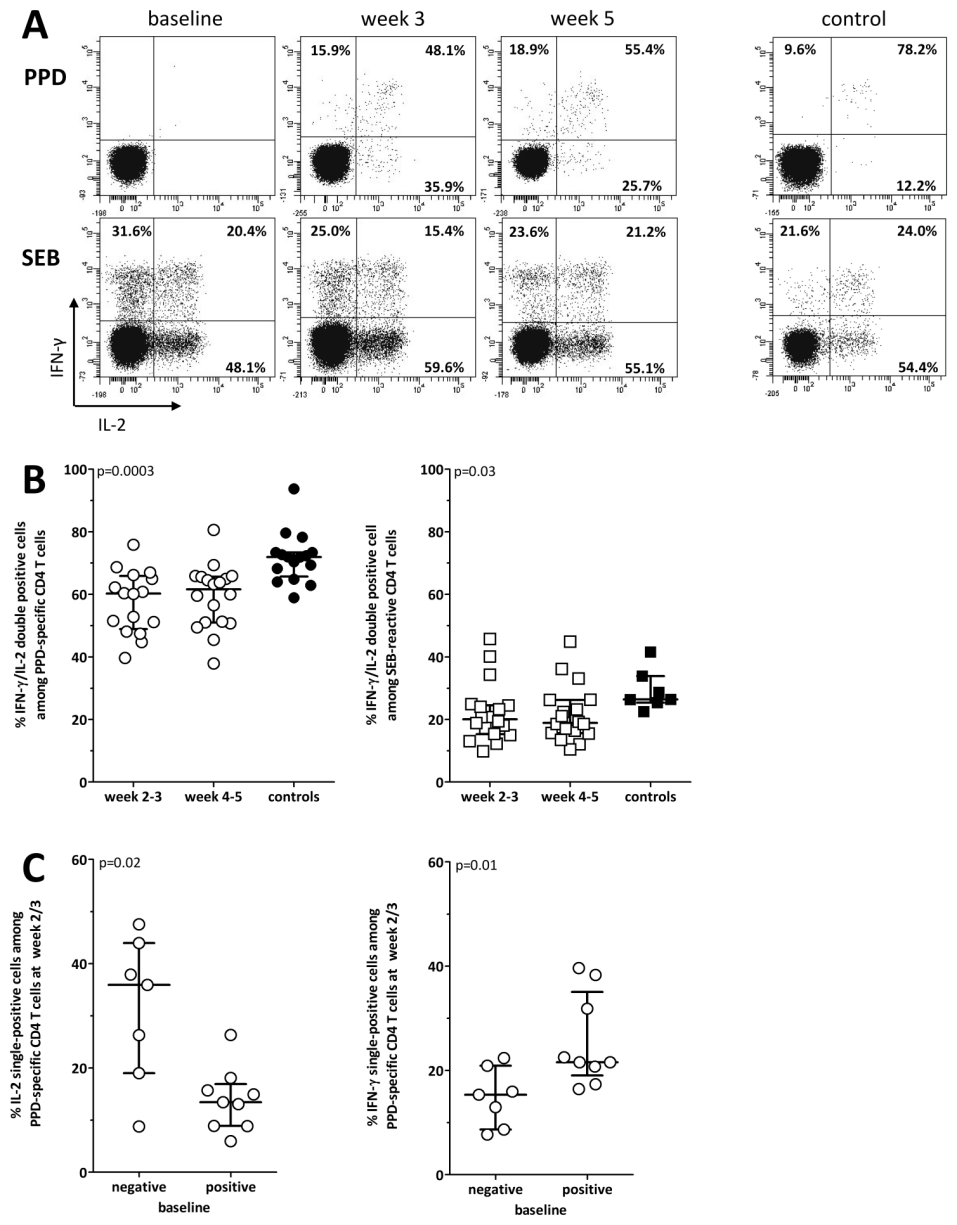
The cytokine-expression profile is altered in patients after repeated BCG-instillation (**A**) Representative dotplots of the flow-cytometric analysis of IFN-γ and IL-2 expression profiles of PPD and SEB-reactive CD4 T cells in a patient before, 3 and 5 weeks after BCG-therapy (same patient as in Figure 2A), and in a healthy control. The percentages shown in the three quadrants refer to the relative percentage of IFN-γ single-positive, IFN-γ/IL-2 double-positive and IL-2 single-positive cells among all cytokine-positive CD4 T cells. (**B**) The percentages of IFN-γ/IL-2 double-positive cells among PPD- (circles) or SEB-reactive (squares) CD4 T cells was quantified for all patients in week 2/3 (PPD n=16; SEB n=18) as well as week 4/5 (PPD n=18; SEB n=18) who had sufficient numbers of cytokine-producing cells after specific stimulation (≥20 events). Less than 20 events were measured in 2/9 patients where overall percentage of PPD specific CD4 T cells at baseline was above 0.05%. By week 2/3, all 9 baseline positive patients had event counts above 20, whereas 2/9 baseline negative patients were still below detection limit of 0.05% and had <20 events. All other samples had event counts of more than 20. Cytokine profiling of CD4 T cells was performed in 16 controls after PPD-stimulation (black circles) and in 7 controls after SEB-stimulation (black squares). (**C**) The percentage of IL-2 single-positive and IFN-γ single-positive cells among PPD-specific T cells after week 2/3 was stratified for patients with negative (n=7) and positive PPD-status (n=9) before therapy. In two baseline negative patients the number of cytokine positive T cells was still below 20 events. Medians and interquartile ranges are indicated.

We have previously shown that a decrease in dual-cytokine positivity of PPD-specific T cells is observed in patients with active tuberculosis, whilst a high percentage of IFN-γ/IL-2 double-positive T cells is typical for individuals with non-active states [16]. To examine whether repeated antigen-contact with live BCG is associated with an altered cytokine profile and whether this differs in patients with and without pre-existing immunity, the IFN-γ/IL-2 expression patterns in patients after BCG-instillation were compared to those of PPD-positive controls. To ensure statistically robust analysis of T cell subpopulations, this analysis was be restricted to samples where the overall number of cytokine-producing T cells after stimulation exceeded 20 events above background. Representative dotplots of PPD- and SEB-reactive cytokine profiles before and during therapy of a patient and a control are shown in Figure 4A. In line with recent contact with live BCG, the patient had only 48.1% and 55.4% of IFN-γ/IL-2 double-positive T cells after week 2 and 5, respectively, whereas 78.2% of PPD-reactive T cells were double-positive in the control. In all tested individuals, the percentage of double-positive cells after instillation was significantly lower in patients compared to controls (p=0.0003, Figure 4B). The percentage of double-positive cells among SEB-reactive T cells was also higher in controls, although this difference was less pronounced (p=0.03, Figure 4B). Interestingly, newly induced PPD reactive T cells in patients without pre-existing immunity had a higher percentage of IL-2 single producing T cells as compared to patients with pre-existing immunity (p=0.02, Figure 4C). Conversely, PPD-specific immunity in the latter showed a shift towards IFN-γ single-positive T cells (p=0.01, Figure 4C). Together this indicates that the repeated BCG-contact is associated with changes in cytokine profiles similar to patients with active tuberculosis. A shift towards IL-2 single-positivity is typical for patients with primary immunity, whereas a shift towards IFN-γ single-positivity seems to be associated with immunity after reactivation.

### Pre-existing cellular immunity against PPD does not seem to predict treatment success

**Figure 5:**
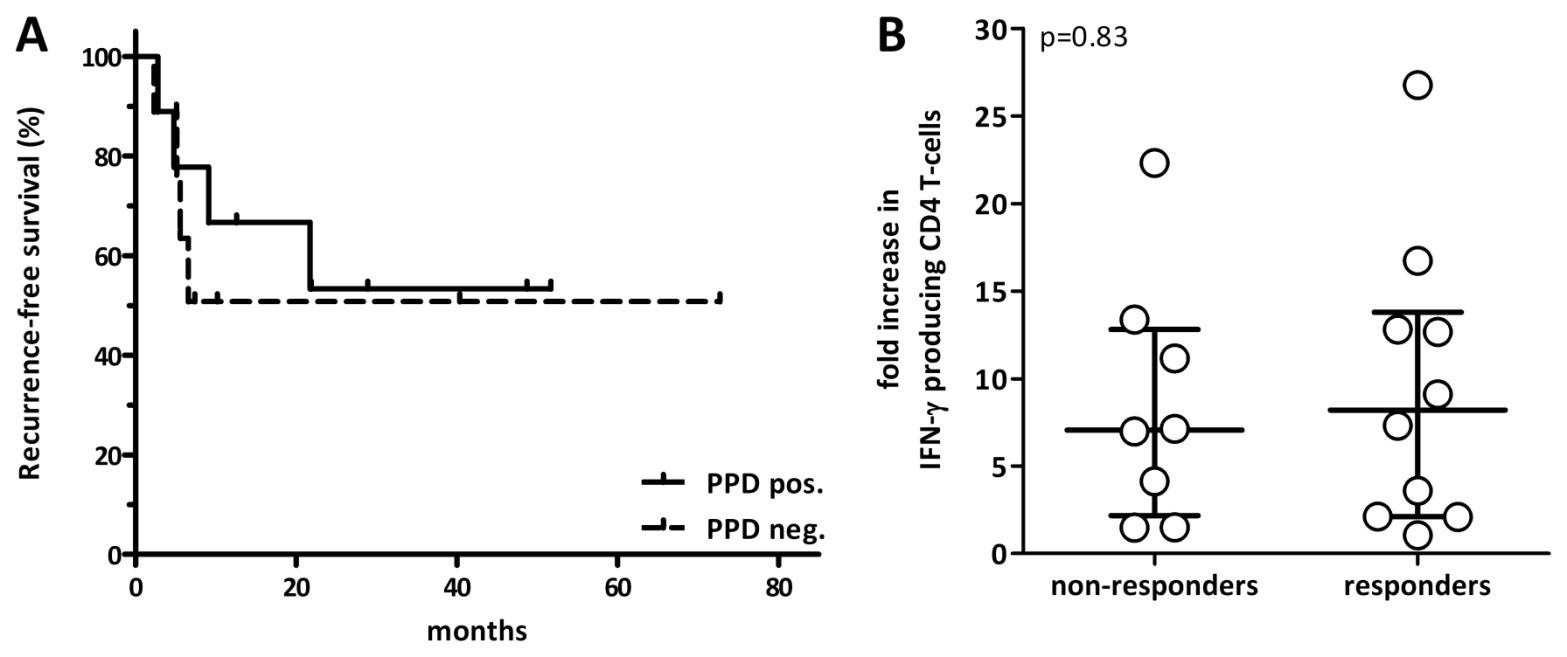
No measurable effect of systemically induced PPD-response on treatment success. (**A**) Recurrence-free survival depending on PPD-status before therapy. (**B**) The increase of percentages of IFN-γ producing CD4 T cells among all specific T cells after stimulation with PPD between baseline and maximum value was compared in non-responders (white circles, n=8) and responders (black circles, n=10).

Finally, although this study is limited by a small sample size and a heterogeneous maintenance therapy, a preliminary analysis of pre-treatment PPD-reactivity and PPD-specific T cell increase in relation to treatment success was performed. Eight patients were classified as non-responders (mean time to relapse 7.5±5.9 months) and 10 were responders (mean follow-up 30±22.7 months). The presence of PPD-specific T cells at baseline does not seem to be related to subsequent treatment success, as baseline PPD-specific immunity, measured by IFN-γ reactive cells, was present in 4 out of 8 non-responders and 5 out of 10 responders (p=1.0), and there was no difference in recurrence-free survival in both groups (Figure 5A). Similarly, the extent of PPD-specific T cell increase did not predict treatment success, as the increase in IFN-γ reactive CD4 T cell frequencies after BCG instillation was 7.1 fold (IQR 10.7) in non-responders and 8.2 fold (IQR 11.7) in responders (p=0.83, Figure 5B). Thus, an effect of pre-existing immunity or systemic increase in PPD-reactive T cells after BCG-induction on treatment success was not discernable in the context of this study.

## Discussion

Instillation of live BCG-bacilli is an effective therapy to prevent progress of non-muscle invasive bladder cancer [26]. In this study, we have used intracellular cytokine staining to characterise the induction of PPD-specific T cell immunity before and during BCG-treatment. We show that a standard BCG-induction course induces a systemic PPD-specific T cell response that increases in magnitude over time, and is clearly detectable in all patients after 4-5 weeks of therapy. Consistent with findings from immunity towards other pathogens or vaccines [27,28], both the induction kinetics and the cytokine profiles differed depending on pre-existing immunity towards PPD. As the immune response profile after instillation of live BCG-bacilli appears to be similar to that in active infection with *M. tuberculosis* [16,29], this therapy may represent a viable model to longitudinally study the induction of antigen-specific immunity before and after natural infection with *M. tuberculosis* in humans, where knowledge on is limited by the fact that the time of infection is rarely known.

Our observations in patients without pre-existing immunity illustrates that the *de novo* induction of a systemic T cell response towards PPD is only observed 2-3 weeks after the first instillation. This delayed time course is typical for slowly replicating bacteria, and similar to that of T cell immunity after natural *M. tuberculosis* infection or after vaccination which takes several weeks for induction [30,31]. Consistent with active replication of BCG-bacteria, the cytokine-expression profile of BCG-induced T cells in both patients with and without pre-existing immunity exhibited a decrease in dual-positivity which is similar to our observations in patients with active tuberculosis [16]. Of note, despite this uniform decrease in dual-positive T cells in patients with active tuberculosis, it was unclear, why the cytokine profile in some patients showed a shift towards IFN-γ and others rather shifted towards IL-2 [16]. The results from this study now show, that this seems to be related to the presence or absence of pre-existing immunity, as a higher percentage of newly induced T cells in patients without pre-existing immunity was shifted towards IL-2 single-positivity. This is consistent with a dominance in IL-2 positive PPD-specific T cells over IFN-γ positive cells that was recently described in infants early after BCG vaccination [31]. This cytokine profile also resembles that of acutely resolving infections and may sustain proliferative activity to ensure expansion of specific immunity [27]. Interestingly, BCG-induced immunity in patients with pre-existing immunity rather exhibited a shift towards IFN-γ single-positivity, which is consistent with cytokine profiles of antigen-specific T cells during reactivating infections such as cytomegalovirus, that is also characterised by a decrease in IFN-γ/IL-2 double-positivity and a concomitant shift towards IFN-γ single-positive cells [32]. These observations may also be relevant for patients with active tuberculosis or their contacts where the pre-infection status is rarely known. In this situation, the difference in cytokine profiles of *M. tuberculosis*-specific T cells may be used to distinguish individuals with primary infection from reactivation and hence may be helpful in tracing natural history of *M. tuberculosis* infection.

PPD-specific T cell responses show considerable interindividual differences at a given time point before and after instillation. This is illustrated by the fact that T cell levels that were reached 4-5 weeks after instillation in patients without pre-existing immunity were in the same range as baseline levels in patients with pre-existing immunity. As the proliferative capacity was shown to increase during BCG-therapy [10], this implies that sufficient T cell levels may be reached earlier and require fewer instillation cycles in individuals with pre-existing immunity. The particular role of pre-existing immunity for shortening the time from instillation to tumor infiltration is supported by a recent study in mice that convincingly showed that parenteral pre-immunisation with BCG not only accelerated tumor infiltration but also therapeutic efficacy [9]. Although systemic immunity may also be assessed using skin-testing or proliferation assays [33,34], the flow-cytometric assay may be performed within one day and used in a clinical setting for an individualised monitoring of the induction of a specific immunity. This approach could also be used to explore the efficacy of other instillation strategies with reduced dosages or fewer cycles, and thereby contribute to a reduction in toxicity.

Murine models have indicated that systemic immunity after BCG-instillation may serve as estimation for the amount of T cells with therapeutic potential at the site of tumor [9]. Evidence for tumor-infiltrating T cells and its association with response to therapy in humans has also been shown by immunohistochemistry from biopsies [35], but this is difficult to perform as screening tool on a routine basis. However, a positive skin-test may be considered as an inflammatory response based on T cell infiltrates at the site of cutaneous induration, and skin-test induration generally correlates with PPD-reactive T cells analysed from whole blood [23,25]. Hence, many studies in humans have therefore aimed at associating skin-test conversion with long-term outcome after therapy, but results were heterogeneous [36,37]. Although our study was not powered for robust outcome analysis, neither pre-treatment frequencies nor induction kinetics of PPD-specific T cells showed any discernable association with long-term treatment outcome. Apart from the low sample size, several reasons may account for this. In terms of outcome analysis, our study was limited by the fact that systemic immunity was only assessed during induction course. Considering the particular role of maintenance therapy [38,39], future studies with larger sample sizes should also include its effect on the maintenance of systemic immunity and its correlation to therapy response. In addition, treatment outcome may be determined by the fact that T cells from whole blood may have variable potential for homing to sites of inflammation. Interestingly, this may be associated with age, as aged individuals show a decreased T cell entry into the skin after tuberculin skin-testing, and hence a decreased cutaneous T cell immunosurveillance [40,41]. As this was mechanistically linked to a decreased TNF-α secretion by local macrophages [41], this may similarly reduce T cell infiltration in response to other types of inflammatory reactions such as that induced by BCG-instillation in the bladder.

Taken together, flow-cytometric analysis of PPD-specific T cells in patients after BCG-instillation was used as a model to longitudinally study the quantitative and functional properties of PPD-specific T cells before and after challenge with mycobacterial antigens. As in patients with active tuberculosis, repeated antigen contact was associated with a decrease in IFN-γ/IL-2 double-positive T cells, and a shift towards IFN-γ single or IL-2 single-positivity distinguish patients with reactivation from those with primary immunity, respectively. Larger studies are required to assess the role of systemically induced immunity for treatment outcome. Given the particular role of T cell infiltration for treatment success, the same technology may be applied with bladder wash samples (unpublished observations, and [42]) to further study the accumulation of PPD-specific T cells at the site of instillation as an additional biomarker for treatment outcome in patients after BCG-instillation therapy.
